# The experience of and coping with an induced abortion: A rapid review

**DOI:** 10.4102/hsag.v26i0.1543

**Published:** 2021-06-30

**Authors:** Roché Lyon, Karel Botha

**Affiliations:** 1 Department of Psychology, Faculty of Health Sciences, North-West University, Potchefstroom, South Africa

**Keywords:** induced abortion, stressors, coping, stigma, social context

## Abstract

This rapid review was conducted to determine the scientific evidence available on how women experience induced abortion and how they cope with the subjective experience thereof. The aim of this review was to systematically explore and synthesise scientific evidence on how women experience and cope with induced abortion. The guidelines of the National Institute for Health and Clinical Excellence were used as a framework to review current international and national literature. The researchers made use of Ebsco Discovery Service to search for relevant studies. This was done by employing key concepts and related synonyms. Eleven relevant studies were identified. As the study was exploratory in nature, covering a relatively small selection of studies, heterogeneous in methodology and cultural focus, only a few general trends were highlighted. Not much information was found for women in the South African context. The study found that socio-economic disadvantages and premorbid relationships are important factors that should be better researched, understood and managed in a South African context. Despite many studies on how women experience and cope with induced abortion, the review revealed the need for research related to specific challenges and experiences of South African women.

## Introduction

The most common surgical procedure performed on women of child-bearing ages is known as induced abortion (American College of Pediatricians 2018). Induced abortions because of unintended and unwanted pregnancies occur in all societies regardless of medical, financial, educational or religious status (Torriente, Joubert & Steinberg 2016).

This study will focus on induced abortion because of an unwanted pregnancy and not on spontaneous abortion or miscarriage or abortion that takes place in order to prevent damage to the mother’s health. The term ‘abortion’ will therefore refer to legal induced abortion from here on. In South Africa, 73 072 abortions were performed in legal state health facilities in 2017 (Bhekisisa Mail & Guardian Centre for Health Journalism 2018).

Despite a notable increase in the number of studies on abortion, contradictory evidence is presented on how women experience and cope with abortion. As argued by Suffla (1997) and Thobejane (2001), there is no painless way of dealing with an unwanted pregnancy. South African research indicates several challenges, including moral conflict and negative emotions (Mojapelo-Batka & Schoeman 2003), regret, guilt, self-blame, judgement and physical pain after abortion (Mookamedi, Mogotlane & Roos 2015). However, a decision to undergo an abortion may be viewed by some as a means of resolving the stress associated with an unwanted pregnancy: it may lead to relief rather than negative psychological experiences or long-term mental health problems (Major et al. 2009). It is also possible that women may not necessarily become depressed, nor experience short- or medium-term trauma (Subramaney et al. 2015). However, evidence presented in a systematic review conducted by the National Collaborating Centre for Mental Health (NCCMH 2011) in the United Kingdom indicates significant limitations pertaining to the relationship between unwanted pregnancy, abortion and mental health.

According to the American College of Paediatricians (2018), research on abortion is often accompanied by research bias. Many researchers who are in favour of abortion seem to downplay the consequences of abortion, whilst those who oppose abortion tend to emphasise the consequences of abortion. Charles et al. (2008) indicated that whilst studies with flawed methodologies tend to find negative mental health sequelae of abortion, studies of a higher quality suggest few, if any, mental health differences between women who had abortions and their respective comparison groups. In spite of the suggestion by Robinson et al. (2009) that the most reliable predictor of post-abortion health is mental health prior to abortion, it seems that we do not have a clear and scientific understanding of the experience of abortion or of factors that influence the long-term mental health outcomes thereof.

Furthermore, no clear South African data are available on how women subjectively experience abortion and cope with it. Lazarus and Folkman’s (1984:141) classic definition states that coping includes ‘constantly changing cognitive and behavioural efforts to manage specific external and/or internal demands that are appraised as taxing or exceeding the resources of a person’. More recently, coping has been referred to as the ability to mobilise, modulate, manage and coordinate one’s behaviour, emotions and attention under stress (Skinner & Zimmer-Gembek 2009). These perspectives imply that coping is a dynamic process of stress management, rather than a passive response from the individual. Taking this into account, the question this study aims to address is as follows: *what broad themes can be identified from available research on the experience of and coping with abortion, and to what extent does available research reflect the dynamic nature of coping*? This research could indicate a gap in research, specifically focused on the South African context. It could also provide directions for further research, whilst indirectly assisting health service providers in South Africa with the following: (1) a better understanding of women’s experience of abortion, (2) discussing and directing pregnancy options, (3) counselling and (4) referrals for post-abortion care.

The aim is therefore to systematically explore and synthesise scientific evidence on how young adult women experience and cope with induced abortion.

## Methodology

A rapid review of current international and national literature on the experience of abortion and ways of coping with the experience of abortion was conducted. A rapid review is an accelerated or streamlined systematic review (Ganann, Ciliska & Thomas 2010). It was specifically decided to do a rapid review as this was the first of three phases in a larger study, and as it could be a useful precursor for further research (Petticrew & Roberts 2006) based on the findings of an exploratory study like this. The guidelines of the National Institute for Health and Clinical Excellence (NICE 2012) for conducting a rapid review were used as a basic framework for this study. This rapid review aimed to answer the following two research questions: (1) *how do young adult women experience induced abortion*? and (2) *how do young adult women cope with the experience of induced abortion*?

### Search strategy

The OneSearch portal, also known as Ebsco Discovery Service, which is a simple search engine that provides a fast, accurate and comprehensive search of 262 electronic databases, was used to search for relevant studies. These electronic databases included, for example, JSTOR, Medline and PubMed. Keywords were selected by applying the key concepts and related synonyms. The following keywords were used in combination with Boolean operators (AND, OR, NOT) to conduct the search:

In the abstract:

Cope OR Coping OR manag* OR adapt* OR adjust* OR handl* OR surviv* OR endur* OR control* OR ‘proactive coping’ OR ‘reactive coping’ OR ‘emotion focused coping’ OR ‘stress management’ OR ‘problem focused coping’ OR experience* OR ‘living with’.

AND (in title):

Abortion* OR ‘Termination of pregnancy’ OR ‘abortion*’ OR ‘elective abortion*’ OR ‘therapeutic abortion*’ OR feticide* OR aborticide* OR ‘deliberate miscarriage*’ OR ‘unplanned pregnanc*’ OR ‘unwanted pregnanc*’ OR ‘legal abortion*’ OR abortifacient* OR ‘unintended pregnanc*’ OR feticide* OR ‘induced miscarriage*’ OR ‘medical abortion*’ OR postabortion* OR ‘abortion trauma’ OR ‘post-abortion syndrome’.

AND (in abstract):

‘Young adult*’ OR ‘emerging adult*’ OR ‘college student*’ OR ‘university student*’ OR student* OR ‘18–25’ OR ‘young women’.

All published English empirical studies, qualitative, quantitative, mixed- or multimethod in design, were included with no limit on the date of publication, as the aim was to gather as much information as possible. Studies had to focus on coping with induced abortion because of an unwanted pregnancy amongst women aged 18–25 years. Review studies, unpublished studies, conference proceedings and studies in a language other than English and without an abstract in English were excluded.

**FIGURE 1:**
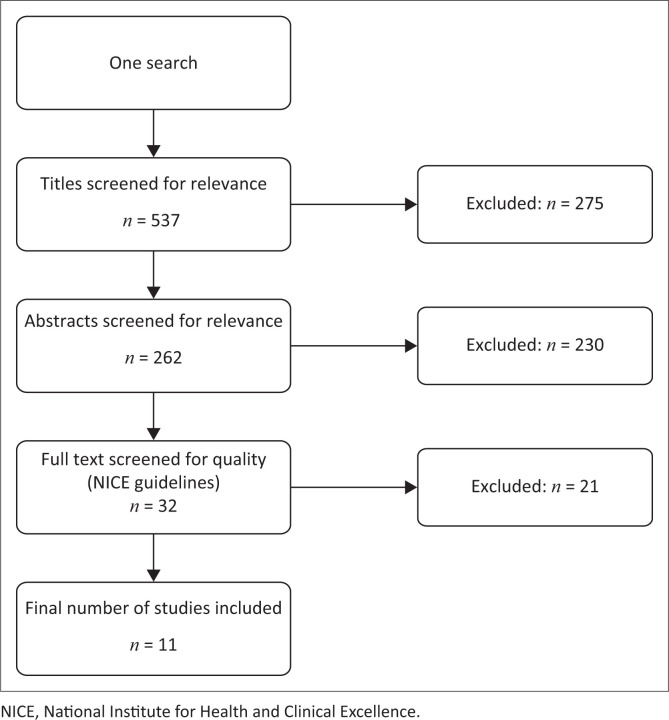
Search flow chart: Realisation of search strategy.

The two researchers, independently of each other, screened studies for relevance based on title only (*n* = 537). Thereafter, a second screening was done based on abstracts (*n* = 262). Figure 1 represents a schematic overview of the screening process. Full texts of remaining studies (*n* = 32) were assessed for scientific quality according to the assessment tools for both quantitative and qualitative studies (NICE 2012) to identify the final 11 studies for inclusion.

The framework for the Evidence for Policy and Practice Information and Coordinating Centre (EPPI 2007) for conducting systematic reviews was used to extract the data from the 11 selected studies (see Table 1). The framework is based on a standardised set of predetermined criteria for each study with reference to: (1) general descriptive information, (2) research methodology and (3) results.

**TABLE 1:** Data extraction of 11 eligible studies.

Number	Author(s) and title	Method	Sample characteristics	Measurement	Core findings
1	Boulind & Edwards (2008). ‘The assessment and treatment of post-abortion syndrome: A systematic case study from South Africa.’	Qualitative	One female, black, Zimbabwean, 22 years old	Intake interviews, session records, beck depression inventory II (BDI-II), beck anxiety inventory (BAI).	Suppression of event and lack of support contributed to PTSD. Experienced range of negative emotions, but also relief when abortion was disclosed.
2	Costa et al. (1987). ‘Psychological correlates and antecedents of abortion: an exploratory study.’	Quantitative	Longitudinal study: 215 high school women (aged 16–18 years); young adults (average age 22 years).	Questionnaire: Scales/indexes to assess personality, social and behavioural variables within framework of problem-behaviour theory.	Premorbid factors influence experience. Women who have had abortion tend to be more unconventional.
3	Curley & Johnston (2013). ‘The characteristics and severity of psychological distress after abortion amongst university students.’	Quantitative	151 female students: 89 who underwent abortions (aged 18–35 years); single, non-child-bearing; had never been pregnant and reported no other stressful life events.	Brief symptoms inventory (BSI), beck depression inventory (BDI), state-trait anxiety inventory (STAI), impact of event scale (IES), perinatal grief scale (PGS) and demographic and health questionnaire	Psychological distress after abortion is multifactorial, associated with co-existing mental health problems and overall emotional health.
4	Fergusson et al. (2006). ‘Abortion in young women and subsequent mental health.’	Quantitative	1265 children born in New Zealand to women for whom information on pregnancy history and mental outcomes were available. Sample sizes ranged between 506 and 520. Age range: 15–25 years.	Interviews using DISC and CIDI.	Increased risk of concurrent and subsequent mental health problems, including depression, anxiety, suicidal behaviour and substance abuse disorders.
5	Fingerer (1973). ‘Psychological sequelae of abortion: anxiety and depression.’	Quantitative	Group 1a: Women visiting abortion clinic, 14–44 years old. Group 1b: 177 abortion patients. Group 2: 207 men and women who accompanied group 1a to clinic. Group 3: 21 male and 15 female postdoctoral students in psychology. Group 4: 43 females.	State-trait anxiety inventory-state (STAITS). Affective adjective check list-today (AACL). Depressive symptomatology (SDS).	No immediate anxiety after abortion. Psychological after-effects seem to reside in psychoanalytic discomfort. Social stigma contributes to socially learned responses to abortion. Abortion seems to result in immediate relief.
6	Gray (2015). ‘“It has been a long journey from first knowing”: Narratives of unplanned pregnancy.’	Qualitative	241 university students; women aged 18–24 years who experienced an unplanned pregnancy. A total of 46 of the 241 terminated their pregnancies and responded to the open-ended narrative question.	Online, semi-structured survey with open-ended narrative question.	Experience range of negative emotions. Narratives effective in positively reappraising and making sense of the experience.
7	Halldén et al. (2009). ‘Early abortion as narrated by young Swedish women.’	Qualitative	10 women aged 18–20 years after medical or surgical abortion in the 6th to 12th week of pregnancy.	Narrative interviews.	Multitude of complex meanings: Protectiveness/responsibility; thoughtful decision making; imagining loss of child-to-be; ambivalent feelings; experience of pain and injustice; relief.
8	Harden & Ogden (1999). ‘Young women’s experiences arranging and having abortions.’	Qualitative.	54 young women between ages 16 and 24 years who had an abortion.	Interviews.	Most experiences positive; negative experiences compensated for by supportive staff. Some experienced judgement and insensitivity from health professionals. Abortion brought relief and restored lack of control.
9	Lundell et al. (2013). ‘Post-traumatic stress amongst women after abortion: A Swedish multi-centre cohort study.’	Quantitative.	1457 women who requested abortion, aged 15–35 years and older.	Screen questionnaire-posttraumatic stress disorder (SQ-PTSD); hospital anxiety and depression scale (HADS).	Few developed PTSD or PTSS after abortion. Experienced guilt, sadness, ambivalence, depression and anxiety. Painful feelings decreased.
10	Monsour & Stewart (1973). ‘Abortion and sexual behaviour in college women.’	Qualitative.	20 single young college women, aged 18–22 years. A total of 14 of the 20 earned a family income of $10 000 and above	Interviews and demographic questionnaire.	No appreciable psychological after-effects. Abortion brings relief from stress; resolves crisis of unwanted pregnancy. Evidence of psychological growth.
11	Taft & Watson (2008). ‘Depression and termination of pregnancy (abortion) in a national cohort of young Australian women: The confounding effect of women’s experience of violence.’	Quantitative	14 776 women, aged 18–23 years in survey 1; 9683 women aged 22–27 years in survey 2.	Centre for Epidemiological Studies depression scale (CES-D). Survey included socio-demographic variables; questions on reproductive events; three questions about violence.	Partner violence, social disadvantages, significantly impacted on women’s depression.

PTSD, post-traumatic stress disorder; DISC, Diagnostic Interview Schedule for Children; CIDI, Composite International Diagnostic Interview; PTSS, post-traumatic stress symptoms.

### Data analysis

A narrative synthesis (Popay et al. 2006) was conducted to synthesise the findings from the 11 studies. This is a trustworthy approach often used in rapid reviews to synthesise the findings from multiple studies (Popay et al. 2006). The data were analysed according to the guidelines provided by Petticrew and Roberts (2006).

### Ethical considerations and trustworthiness

The Health Research Ethics Committee (HREC) of the North-West University (NWU) provided ethical approval for this research (ethics approval number NWU-00059-16-S1). A rapid review is rigorous and systematic and adheres to the core principles of a traditional systematic review to avoid bias during any stage of the process (Schünemann & Moja 2015). The researchers adhered to a carefully planned research process by strictly following the NICE and EPPI guidelines. The research was conducted in a reflective way, whilst all results were recorded and documented according to the review protocol. During continuous reflection on the research process care was taken to ensure that the research was both thorough and critical. Finally, trustworthiness and credibility were increased by adhering to the three guidelines proposed by Wager and Wiffen (2011:131–133), namely to avoid redundant publication, plagiarism and ensure transparency.

## Results

Eleven studies published between 1973 and 2015 were identified as eligible for inclusion (see Table 1). Of these, six studies used quantitative methods (Costa, Jessor & Donovan 1987; Curley & Johnston 2013; Fergusson, Horwood & Ridder 2006; Fingerer 1973; Lundell et al. 2013; Taft & Watson 2008) and five used qualitative methods (Boulind & Edwards 2008; Gray 2015; Halldén, Christensson & Olsson 2009; Harden & Ogden 1999; Monsour & Stewart 1973). Quantitative methods almost exclusively included questionnaires and standardised scales, whilst qualitative data were primarily collected by means of in-depth interviews.

The majority of the studies focused on the mental health sequelae of unplanned pregnancy and abortion with different focuses, namely, general mental health (Fergusson et al. 2006; Harden & Ogden 1999), general stress and depression (Curley & Johnston 2013; Taft & Watson 2008), post-traumatic stress (Boulind & Edwards 2008; Lundell et al. 2013) and sexual self-image (Monsour & Stewart 1973). One of the studies was carried out in South Africa (Boulind & Edwards 2008), four in the United States of America (Costa et al. 1987; Fingerer 1973; Gray 2015; Monsour & Stewart 1973), two in Sweden (Halldén et al. 2009; Lundell et al. 2013), one each in the United Kingdom (Harden & Ogden 1999), Australia (Taft & Watson 2008) and New Zealand (Fergusson et al. 2006) and one study in both Canada and the United States of America (Curley & Johnston 2013).

The identified themes are presented according to the two aims of the research study, namely (1) young adult women’s experience of induced abortion and (2) the coping strategies of young adult women who experienced induced abortion.

### Young adult women’s experience of induced abortion

Three subthemes emerged from the reviewed articles: (1) the effect of premorbid factors on the abortion experience, (2) stressors related to the abortion and (3) negative emotions experienced in relation to the abortion.

#### The effect of premorbid factors on an abortion experience

Taft and Watson (2008) found both premorbid poverty or social disadvantages disadvantages and partner violence to have an effect on a woman’s experience of abortion. This finding relates to post-abortion depression specifically and is integrated later when negative experiences related to abortion are discussed. Costa et al. (1987) indicated personality as a premorbid factor, specifically related to the decision to abort or not. Women who decided to abort appeared to be more unconventional and less conforming to socially accepted norms and behaviour than those who did not. These women were also more socially critical and more liberal regarding sex roles and politics, less tolerant of deviance, less religious and had fewer moral objections to abortion.

These authors measured unconventionality based on personal beliefs that include social criticism, sex role and political liberalism and personal controls that include intolerance of deviance, religiosity, and a specific moral attitude regarding abortion. Therefore, the decision to have an abortion is related to a pre-existing pattern of unconventionality. Age-related issues did not emerge as a factor at all.

#### Stressors related to abortion

Making the decision to undergo an abortion is a stressful event in itself. This section discusses three stressors related to abortion found in the reviewed literature. These stressors include (1) social stressors, (2) ambivalence and (3) lack of autonomy and control.

**Social stressors:** Fingerer (1973) found that the post-abortion psychological discomfort that women experienced is because of socially learned responses to the abortion situation rather than as a result of the abortion itself. These responses specifically relate to the ‘motherhood myth’, namely, that a woman’s highest function is to be a ‘good mother’, which is a social construct stating that a woman has not fulfilled her highest function when she undergoes an abortion. Not only do socially learned responses play a role in the abortion experience, but the social reactions from hospital staff, for example, judgement and insensitivity (Harden & Ogden 1999; Monsour & Stewart 1973), also caused women to experience more stress. Furthermore, there may be a link between the abortion stigma and social support – one of the participants in Gray’s study (2015) reported that she kept her abortion a secret because of fear of the social stigma attached to abortion. Lack of social support is a further factor indicated in the reviewed literature (Boulind & Edwards 2008; Gray 2015; Monsour & Stewart 1973), which relates to secrecy, avoiding the abortion stigma and experiencing rejection from unsupportive friends.

**Ambivalence:** Three studies (Boulind & Edwards 2008; Gray 2015; Lundell et al. 2013) reported that women experienced ambivalence about the abortion decision. Halldén et al. (2009) specifically found abortion to be a complex event characterised by the experience of ambivalent positive and negative emotions.

**Lack of autonomy and control:** Three studies (Curley & Johnston 2013; Gray 2015; Harden & Ogden 1999) reported specific stressors related to various aspects of the study participants’ lives. According to Curley and Johnston (2013), the feeling of being overwhelmed by the unplanned pregnancy and the abortion was a main stressor, which subsequently developed into different mental health issues. Gray (2015) stated that the most recurrent and dominant stressors that women experienced throughout the abortion involved lack of control over one’s body and inner self. Lack of control over one’s body was also found to be a major stressor by Harden and Ogden (1999). They further observed that the prolonged abortion procedure contributed to additional stress, together with a strong desire for things to return to normal.

#### Negative emotions experienced in relation to abortion

Apart from social trauma, women also experienced painful emotions on an individual level. These emotions, namely (1) depression, (2) anxiety, (3) physical and emotional pain and (4) guilt, are discussed in the following sections.

**Depression:** Fingerer (1973) found an occurrence of mild depression, most likely because of the reactive situational adjustment in the form of desensitisation or re-evaluation of learned beliefs regarding abortion. According to Boulind and Edwards (2008), depression developed when thoughts and feelings associated with the abortion were repressed. Depression was further worsened by feelings of guilt and other factors unrelated to the abortion. According to Gray (2015), women experienced feelings of helplessness and depression.

Taft and Watson (2008) provided a slightly different explanation and indicated that abortion only has a peripheral association with depression. According to them, 30% of women between the ages of 22 and 27 years were likely to have depression after abortion, but they argued that both poverty and social disadvantages contribute to depression also. Women who were married or living with their partners were less likely to report depression than single or divorced women. Women with post-secondary qualifications, tertiary degrees and private health insurance were less likely to be depressed when compared with women with secondary education and women with no private health insurance. In addition, women with clerical or trade jobs or with home duties were also more likely to be depressed than managerial or professional women. However, and perhaps surprisingly, women living in rural areas were less likely to feel depressed compared with women living in urban areas. Women who were exposed to partner violence were significantly more likely to experience depression after abortion, which is further enhanced by other adverse reproductive events, namely, more frequent pregnancies, miscarriages and troubled reproductive history (Taft & Watson 2008). According to Taft and Watson (2008), these factors rather than their experience of the abortion itself increase the possibility of experiencing depression.

**Anxiety:** Fingerer (1973) reported that no immediate anxiety was observed after the abortion. However, Fergusson et al. (2006) found that women who had abortions experienced higher rates of anxiety disorders than women who did not have abortions. One of the studies (Gray 2015) reported that women experienced anxiety for different reasons, namely, fear of being exposed to others because of the abortion, anxiety about the future pregnancy and having to deal with the abortion on their own. Curley and Johnston (2013) measured state and trait anxiety amongst three groups of women, namely, women who had an abortion and preferred psychological services, women who had an abortion and did not prefer psychological services and a control group, and found that more than 50% of women who had an abortion preferred psychological services, whilst situational anxiety was higher in this group than in the other groups. Boulind and Edwards (2008), using the Beck Anxiety Inventory (BAI) in a case study, reported panic, generalised anxiety and residual symptoms of anxiety 3 months later. Harden and Ogden (1999) found that women experienced anxiety in the form of isolation and disempowerment. Furthermore, Lundell et al. (2013) observed that although anxiety is naturally associated with abortion, only a minority of women developed post-traumatic stress disorder (PTSD) or even post-traumatic stress symptoms (PTSS) after abortion. However, these symptoms were because of trauma experiences unrelated to the abortion.

**Physical and emotional pain:** Studies carried out by Gray (2015) and Halldén et al. (2009) reported that participants described the abortion procedure as both physically and emotionally painful, whilst Curley and Johnston (2013) reported that physical pain may have led to anxiety. Halldén et al. (2009) specifically reported that the emotional pain was associated with observing the foetus during the abortion and losing a child that could have lived. A participant in the study conducted by Boulind and Edwards (2008) expressed pain because of her isolation and lack of support throughout the abortion process.

**Guilt:** In the study performed by Lundell et al. (2013) it was found that only a small group of women experienced guilt arising from feelings of ambivalence about the abortion. The women in Gray’s study (2015) constantly reflected on the abortion because of their guilt feelings about their decision. They reported feeling like a bad person, experienced guilt about having unprotected, irresponsible sex and regarding future pregnancies and motherhood. Harden and Ogden (1999) also found that participants reported feelings of guilt about falling pregnant and irresponsible sexual behaviour. A participant in the study performed by Boulind and Edwards (2008) mentioned that she felt guilty about the abortion because of shame, fear of disappointment and feeling like a ‘bad’ person.

### Coping strategies of young adult women who experienced induced abortion

Two subthemes with regard to young women’s coping strategies were identified, namely (1) coping efforts and strategies and (2) psychological growth gained with the abortion experience.

#### Coping efforts and strategies

The following sections discuss different mechanisms utilised by women to cope with abortion. Different studies show different coping mechanisms such as self-reflection and avoidance.

**Self-reflection:** Trying to make sense of the experience One can employ different ways to make sense of the decision to undergo abortion. As will become clear in the case of self-reflection, women found meaning in a variety of ways. Gray (2015) found that women reflected on the decision-making process by justifying the decision to abort. In their study, Boulind and Edwards (2008) indicated that a participant used self-criticism because she felt disappointed with herself for rushing into the decision to have an abortion without thorough consideration.

Halldén et al. (2009) found that women used self-reflection for the following reasons: (1) to protect and take responsibility for the pregnancy, including surprise and disbelief about becoming pregnant, visualising themselves as someone else experiencing the reality of the abortion and choosing a healthier lifestyle prior to the abortion; (2) to facilitate thoughtful decision-making by interpreting the abortion as selfish and considering the consequences for the child as unselfish; (3) to deal with sensitivity about the approval of others – support was seen as very important, particularly from mothers, but also from partners and professionals; (4) to reflect on the loss of the child-to-be, imagined as a physical void and not being able to have children later in life; and (5) to obtain an independent, comprehensive understanding of the abortion.

**Avoidance coping:** There are cases in which the primary means of coping involved avoiding the abortion experience as indicated by Curley and Johnston (2013). They further explained that some of the participants were incapable of coping with overwhelming thoughts and feelings about the abortion, preferring psychological services. In the case study of Boulind and Edwards (2008), a participant mentioned that she felt guilty about the abortion and coped by repressing her thoughts about the abortion and avoiding talking about the experience. Finally, Halldén et al. (2009) found that pain was eased by avoiding thinking about the child that they could have had, perceiving the foetus as a non-human being, seeing the abortion as a miscarriage and assigning the responsibility of the abortion decision to the staff that performed the abortion.

#### Psychological growth gained with the abortion experience

Some studies have found that women experienced positive emotions regarding their decision to undergo abortion. The most prominent positive experience was one of relief. The studies carried out by Halldén et al. (2009) and Monsour and Stewart (1973) showed that women felt relief because the abortion resolved the crisis of the unwanted pregnancy. Some studies have shown that women were relieved because they were able to continue to live normal lives after the abortion (Halldén et al. 2009; Harden & Ogden 1999). Two studies found that women experienced relief because of the fact that they could disclose their abortion experience (Boulind & Edwards 2008; Halldén et al. 2009). The study carried out by Harden and Ogden (1999) showed that women felt relief because they did not experience the physical symptoms of pregnancy anymore and also because the abortion experience differed from the negative expectations they had. The only other positive experience, namely, a sense of autonomy regarding the decision to abort, was reported by Gray (2015).

## Discussion

It is evident from the review that the experience of abortion differs amongst women and various factors play a role in their experience of the unwanted pregnancy and abortion. The experience of induced abortion included three subthemes: the effect of premorbid factors on the abortion experience, stressors related to the abortion and negative emotions experienced in relation to the abortion. Furthermore, coping with the experience of induced abortion involved coping efforts and strategies and psychological growth gained from the experience of induced abortion These themes are not exclusive, for example, premorbid factors such as social disadvantage, partner violence and stressors related to the abortion, were found to influence the experience of abortion, whilst coping strategies were most often based on the nature of negative emotions experienced. In general, the findings of the review are not surprising as it is primarily supported by other studies within the review and by literature in general, whilst not many contradictory findings were observed.

The first noteworthy implication of the study is that certain premorbid factors may be important in understanding the experience of, and coping with, abortion. Personality was indicated by Costa et al. (1987) to influence the decision to abort or not. However, as this study was carried out three decades ago, and as attitudes about abortion have changed significantly since then, the finding should be interpreted with caution. An important question is, to what extent does a positive attitude towards abortion make the decision and coping process easier? However, even though a positive attitude towards abortion may be a protective factor, the experience of abortion is often negatively influenced by premorbid socio-economic challenges and partner violence (Taft & Watson 2008). This is especially important in the South African context because a lack of financial resources and social support (Ndwambi & Govender 2015; Sullivan et al. 2017; Torriente et al. 2016) are often indicated as the primary reasons for having an abortion. In addition, it seems that a history of partner violence is often associated with a higher rate of unintended pregnancies and abortion requests (Pallitto et al. 2012; Tinglöf et al. 2015), and is related to more stress, anxiety and depression (Tinglöf et al. 2015). The role of premorbid socio-economic factors should therefore be taken into account in abortion counselling, especially in a developing country such as South Africa.

Secondly, the abortion process is associated with a number of stressors that challenge young women on different levels. Perceived lack of support during an unwanted pregnancy and abortion is not a surprise and is confirmed by researchers such as Sa´nchez-Siancas et al. (2018). Stigma, however, is a more complex issue and may overlap with other factors, for example, perceived lack of support. A systematic review about abortion stigma (Hanschmidt et al. 2016) concluded that women who had abortions experience fear of social judgement, self-judgement and the need to keep their abortion a secret. Mookamedi et al. (2015) reported, based on a South African study, that women felt stigmatised after abortion and perceived themselves as ‘murderers’ because of their religious contexts. It is therefore no surprise that stigma often manifests as feelings of guilt, shame, anxiety, secrecy or unease about other people’s beliefs about abortion (Shellenberg & Tsui 2012; Sullivan et al. 2017). The question therefore emerges as to: whether the guilt and anxiety that women experience is because of the abortion itself, or because of contextual factors such as stigma. Experiencing ambivalence, conflict and a lack of autonomy is expected in any adverse event. The fact that the decision to abort is preceded by an unwanted (or untimely) pregnancy further complicates matters because it challenges one’s goals, identity and values. Although ambivalence and lack of autonomy may be related to perceived stigma and lack of social support, none of the studies explicitly reports it as such.

Thirdly, stressors related to abortion often result in emotions such as negative affect or emotional pain, anxiety and guilt. In some cases, these emotions develop into psychological disorders such as depression and even PTSD. It is surprising that none of the studies report on perinatal grief, although it is implied in most cases where depression or emotional pain was reported. Some contradictions or inconsistencies were also observed, for example, whereas depression was reported in several studies. Taft and Watson (2008) reported that there is no causal link between women’s abortion experiences and depression. This contradiction is also observed in the literature not included in this review (e.g. Boersma et al. 2014; Foster et al. 2015; Gomez 2018). The same is observed regarding anxiety. Even though seven studies in the review reported anxiety symptoms, this is not necessarily supported by the literature not included in the review (cf. Foster et al. 2015). The experience of pain and guilt is subjective, physically and emotionally intertwined and largely supported by the literature (cf. Mookamedi et al. 2015; Sullivan et al. 2017). However, there is not enough evidence on whether and how emotional pain and guilt are separate to either feelings of depression or anxiety. It is probably best to regard it as part of the subjective experience of both. Therefore, even though clear signs of distress are reported, the severity thereof, the extent to which it puts women at risk of developing psychological disorders and which women would be at risk still needs more clarity.

Fourthly, the review reveals two broad coping efforts, namely, avoidance and self-reflection. According to Dykes, Slade and Haywood (2011), avoidance coping is used when the memories about the abortion are suppressed by actively trying to forget and avoid thinking about it, whilst self-reflection is used when the decision to abort and thoughts and fears regarding future fertility are rationalised. Avoidance is usually perceived to be a less adaptive type of coping compared with the recognition, processing and expression of emotion (De Ridder & Kuijer 2006). The functionality of coping efforts should always be viewed in context, given the stigma associated with abortion. Avoidance may only give temporary relief. It could also be that avoidance leads women to self-reflect, which in turn may lead to avoidance if the experience of stigma and rejection is emphasised through self-reflection. However, this should be explored in future research. The lack of problem-solving coping is not adequately explained in any of the review studies, but may relate to feelings of depression and anxiety and the lack of social support. It may also be explained by the general belief that problem-solving coping fits controllable situations, whilst emotion-focused coping fits uncontrollable situations (Benyamini 2009). Therefore, future research should explore the possibility that the women in the review studies did not apply problem-solving coping, as they did not perceive their situation as controllable and did not apply more adaptive emotion-focused coping because of the stigma and secrecy of the situation.

Finally, some evidence suggests the presence of positive experiences in the form of relief and a sense of autonomy despite the challenges young women face. Quinley, Ratcliffe and Schreiber (2014) reported that abortion primarily provides relief to women who present for abortion. Not only did the participants in a study conducted by Kero, Högberg and Lalos (2004) report relief after the abortion but abortion is also described as a positive experience regarding mental growth and maturity. Although not much evidence emerged from the review, both a sense of relief and autonomy may represent a form of self-protection against resource loss from a conservation of resources perspective (Frydenberg 2014). It may even facilitate a broadening of resources from the ‘broaden and build’ theory in positive psychology (Fredrickson 2013). According to Fredrickson, even fleeting micro-moments of positive emotions are able to reshape people by setting them on trajectories of growth and building their enduring resources.

### Limitations

The OneSearch portal, also known as Ebsco Discovery Service, does not completely cover all the databases that exist and therefore some articles may not have been included. Relatively few studies (*n* = 11) were included and caution should therefore be exercised when drawing conclusions about the experience of abortion in general. The findings of older studies should be interpreted keeping in mind that the social context has changed considerably over the period (e.g. Harden & Ogden 1999; Monsour & Stewart 1973). However, it is possible that changing attitudes towards abortion and healthcare may reflect in the behaviour of medical staff today.

The studies were quite heterogeneous in methodological approach and quality, specifically with respect to how the experience of abortion was evaluated and regarding the cultural contexts in which they were conducted. It would therefore be impossible to generalise the findings to a South African context.

Finally, studies were generally not well controlled for the influence of external factors on the abortion experience. Only some aspects related to personality and social background were indicated. However, there may be more external factors that we are not aware of.

## Conclusion and future directions

The aim of this study was to systematically explore and synthesise scientific evidence regarding how women experience and cope with abortion. A rapid review was carried out to synthesise the findings of 11 studies complying with the inclusion criteria. Because the study was exploratory in nature and because the relatively small selection of studies was heterogeneous in methodology and cultural focus, caution was taken not to make any specific conclusions but only to highlight a few general trends.

It was clear from the review that abortion is a complex emotional event that should be understood within the context of each individual. It can be concluded that an unwanted pregnancy and abortion are intertwined, and that the experience of abortion is not a one-dimensional experience in the life of a woman with an unwanted pregnancy. Each woman is uniquely acquainted with her abortion decision, experience and aftermath, mainly because the experience and coping efforts are greatly influenced by each woman’s context (including culture) and pre-abortion mental health.

The studies reviewed do not provide much information about women in a South African context. It is suggested that socio-economic disadvantages and premorbid relationships should be considered in future research. Not only does the abortion context differ but the perceived abortion stigma also stands apart for each woman’s unique narrative. One can also question whether women experience a lack of support because of stigma or because of them isolating themselves as part of the abortion process. Psychological growth after having an abortion is therefore a possibility, but not a given, and will inevitably be affected by the unique abortion context of the individual.

Further research may explore the following areas:

- How premorbid socio-economic factors could be integrated in abortion counselling, specifically in South Africa.
- The severity and extent to which distress put women at risk of developing psychological disorders post-abortion.
- The question whether guilt and anxiety are related to the abortion itself or to perceived stigma or rejection by others.
- The nature and dynamics of coping styles and strategies associated with abortion – more specifically, the extent to which stigma, avoidance and self-reflection are related, and if and why problem-solving coping is generally lacking amongst women during the abortion process.
- Factors that facilitate psychological growth in women after abortion, especially in the long run.
- How South African women experience and cope with abortion, and how this compares with research findings from elsewhere.
